# Severity of liver cirrhosis: a key role in the selection of surgical modality for Child-Pugh A hepatocellular carcinoma

**DOI:** 10.1186/s12957-015-0567-9

**Published:** 2015-04-15

**Authors:** Er-lei Zhang, Bin-yong Liang, Xiao-ping Chen, Zhi-yong Huang

**Affiliations:** Hepatic Surgery Center, Tongji Hospital, Tongji Medical College, Huazhong University of Science and Technology, 1095 Jie Fang Da Dao, Wuhan, 430030 China

**Keywords:** Hepatocellular carcinoma, Surgical outcome, Surgical modality, Liver cirrhosis

## Abstract

Hepatocellular carcinoma is the third leading cause of cancer-related death in the world, and cirrhosis is the main cause of hepatocellular carcinoma and adversely affects surgical outcomes. Liver resection, liver transplantation, and local ablation are potentially curative therapies for early hepatocellular carcinoma (HCC). There exists an obvious histological variability of severity within cirrhosis which has different clinical stages. For patients with Child-Pugh B cirrhosis and/or portal hypertension and HCC within Milan criteria, consensus guidelines suggest that liver transplantation is the best treatment of choice; liver resection is widely accepted as first-line treatment for patients with early-stage HCC and preserved liver function; and local ablation is the treatment of choice in patients with small tumors who are not candidates for surgery or can be used as a temporary treatment during the waiting period for transplantation. For patients with compensated cirrhosis or Child A cirrhosis, the selection of surgical modality based on subclassification of cirrhosis remains unclear. This review examines the current status of the selection of surgical modality for hepatocellular carcinoma treatment in cirrhotic patients and aims to emphasize the effects of the severity of cirrhosis on the selection of surgical modality for the treatment of hepatocellular carcinoma.

## Review

### Introduction

Hepatocellular carcinoma (HCC) is the third leading cause of cancer-related deaths worldwide and primarily arises from cirrhosis [1,2]. The reported overall incidence of HCC in patients with cirrhosis is 3.4% to 5.6% [3,4], and the annual incidence of HCC is 1.7% to 3.7% in Western populations [5]. Some studies have demonstrated that subclassifications of cirrhosis are closely related to the incidence of HCC [6,7]. It has been reported that 60% to 90% of HCC patients have associated underlying cirrhosis [5,8]. Therefore, cirrhosis is an important factor that cannot be ignored in the surgical treatment of HCC.

According to the guidelines of the American Association for the Study of Liver Diseases (AASLD) and the Barcelona Clinic Liver Cancer (BCLC) group, the criteria for safe liver resection (LR) include a Child-Pugh A classification, the absence of portal hypertension and normal serum bilirubin [9]. However, these guidelines do not emphasize the importance of underlying cirrhosis. For the majority of HCC patients, obvious histological variability in the severity of the so-called one-stage cirrhosis exists. For compensated cirrhosis or Child-Pugh A cirrhosis, there are also some advanced cirrhosis (for example, F4B-F4C using Laennec scoring system) which is dangerous for resection; therefore, it is important to recognize these differences to make proper surgical decisions in clinical practice [10]. LR, liver transplantation (LT), and local ablation (LA) are considered to be curative treatments for early HCC patients and offer the best long-term outcomes [11,12]. LT is widely accepted as the optimal treatment for patients with underlying cirrhosis and HCC that fulfills the Milan criteria because it can eliminate both tumors and the cirrhotic liver [13]. However, due to organ shortages, high costs, and tumor progression during the waiting period, the application of LT is largely limited [14,15]. LR remains the mainstay curative approach for early HCC. LR yields an overall survival that is comparable to that of LT [16-18] and is also applicable for lesions not indicated for LT. However, the remnant liver with underlying cirrhosis is prone to multicentric *de novo* carcinogenesis after LR, and the long-term outcomes of such HCC patients undergoing LR were significantly worsened by increased severity of cirrhosis [19,20]. Therefore, the severity of cirrhosis might play a key role in determining whether LR or LT is the most appropriate modality for the treatment of early HCC in these cirrhotic patients. Over recent decades, LA has shown satisfactory long-term outcomes when applied to cases with early HCC. LA is accepted as the first-line treatment for HCCs ≤3 cm in diameter in the current guidelines [21,22]. Increasingly, the question of whether the ablation of small tumors (HCC ≤3 cm) can provide long-term outcomes that are comparable to those of resection when the severity of cirrhosis is considered has been raised. Therefore, understanding the potential outcomes of LT, LR, or LA based on the severity of cirrhosis is essential for the individualization of surgical modalities. This review aims to examine the current status of the selection of surgical modality for HCC.

### Cirrhosis and long-term outcomes of LR

With improvements in surgical techniques, evaluations of liver function, and perioperative care, the long-term outcomes of LR for HCC have greatly improved in recent decades. Unfortunately, the long-term outcomes of LR remain unsatisfactory largely because of the high incidence of postoperative recurrence. Tumor recurrence can result from the spread of HCC cells into the remnant liver via the portal vein before or during LR or new foci due to coexisting cirrhosis and other HCC-relevant risk factors [5,23-25]. Some studies have indicated that the long-term outcomes of HCC patients with cirrhosis are significantly worse than those without cirrhosis after LR [20,25-29]. The 5-year overall survival (OS) rate after LR for HCC in patients with advanced cirrhosis is less than 30%, whereas this rate is more than 50% in those without cirrhosis [20,30,31]. Taura and colleagues [20] compared the long-term outcomes of 127 HCC patients without cirrhosis with those of 129 patients with Child A cirrhosis and 37 with Child B cirrhosis according to the oncological Milan criteria. These authors found that the 5-year OS and recurrence rates were 81% and 54% in the patients without cirrhosis, 54% and 78% in the Child A cirrhosis patients, and 28% and 91% in the Child B cirrhosis patients, respectively. These results indicate that coexisting cirrhosis is associated with higher recurrence rates and lower OS rates. Several subsequent studies also revealed that the severity of fibrosis is strongly associated with 5-year survival, which is significantly worsened by increased liver fibrosis severity [26,32]. In recent decades, cirrhosis has only been considered as a ‘present’ or ‘absent’ variable in surgical studies of HCC, and the subclassification of cirrhosis has not been emphasized. In our previous study, cirrhosis was classified into mild, moderate, and severe based on the morphological changes of the liver as evaluated during surgery. The 3-year OS rates of the HCC patients with mild, moderate, and severe cirrhosis after LR were 74.3%, 48.1%, and 26.7%, respectively (*P* = 0.001). The OS rate was significantly worse in patients with severe cirrhosis than in those with mild cirrhosis [33]. A recent study conducted by Kim [19] revealed that the cumulative recurrence rates significantly increase with increased cirrhosis staging as evaluated by the Laennec staging system. In this study, the 3-year cumulative recurrence rates in patients with no cirrhosis, stage 4A cirrhosis, stage 4B cirrhosis, and stage 4C cirrhosis were 21.8%, 42.9%, 68.5%, and 86.7%, respectively (*P* < 0.001). Therefore, the recognition of the presence of varying underlying severity of fibrosis and cirrhosis is critically important in determining the long-term outcomes of LR (Table 1). The Laennec system is based on liver biopsies and was modified from the METAVIR system. This system has been well established for the staging of the severity of cirrhosis and is widely accepted in studies of chronic liver diseases. The potential application of this system in surgical settings deserves further study, particularly regarding the evaluation of the effects of cirrhotic severity on long-term outcomes of LR.

**Table 1:** **Long-term outcomes of LR in recent studies with comparative analyses of cirrhosis status**

**Authors**	**Cirrhosis status**	**Patients (** ***n*** **)**	**Overall survival (%)**	***P*** **value**
Taura *et al*. [20]	Non-cirrhosis	127	81	<0.001
	Child A cirrhosis	129	54	
	Child B cirrhosis	37	28	
Gassmann *et al*. [32]	Normal liver	21	50	0.032
	Fibrosis (Batts system)	27	28	
	Cirrhosis	24	17	
Huang *et al*. [33]^§^	Mild cirrhosis	29	74.3	0.001
	Moderate cirrhosis	29	48.1	
	Severe cirrhosis	19	26.7	
Santambrogio *et al*. [28]	Child A cirrhosis without PH	160	65	0.024
	Child A cirrhosis with PH	63	48	
Kadri *et al*. [29]	Ishak stages 1 to 2	45	68.9	0.09
	Ishak stages 3 to 6	155	56.8	
Roayaie *et al*. [25]	Fibrosis (METAVIR 0 to 3)	38	84	NS
	Cirrhosis (METAVIR 4)	89	63	
Wang *et al*. [26]	Fibrosis (Ishak 1 to 5)	135	73	0.01
	Cirrhosis (Ishak 6)	54	50	
Kim *et al*. [19]^§^	F3-F4B (Laennec system)	82	91.4	0.007
	F4C (Laennec system)	10	70	

The overall survivals indicate the 5-year overall survival rates, except ^§^ which indicates the 3-year overall survival rate. PH, portal hypertension; NS, not significant.

### LR or LT?

Both LR and LT have been proposed as the first-line treatment for early HCC. No waiting time is required prior to LR; thus, the dropout and tumor progression which are associated with the waiting time for LT are avoided. However, LT can eliminate both tumors and the cirrhotic liver, which is prone to *de novo* occurrences of HCC. Therefore, whether LR or LT is the most appropriate modality for the treatment of early HCC remains controversial. Some studies have indicated that, for HCCs that fulfill the Milan criteria in cirrhotic livers, the long-term outcomes of LT are better than those of LR because both the tumor and the underlying cirrhotic liver are removed [34-36]. Sapisochin and colleagues reported that the 10-year OS rate was 49% among a group of LT patients and that this rate was 33% in an LR group. In this study, the 10-year recurrence rates were 20% and 83% for LT and LR, respectively (*P* < 0.01) [34]. However, other studies have suggested that LR can achieve 5-year OS rates that are similar to those of LT [16,37-39]. Two meta-analyses compared the long-term outcomes of LT and LR for HCCs that fulfilled the Milan criteria and came to opposing conclusions [40,41]. Notably, tumor progression and dropout during the waiting period might artificially improve the long-term outcomes of LT. Intent-to-treat (ITT) analysis, which accounts for tumor progression and dropout among potential LT patients, has been proposed for comparing survival between LT and LR. Koniaris compared the long-term outcomes of LR with those of LT using an ITT analysis and found that the 5-year OS rates were not significantly different between LR and LT for HCC patients who fulfilled the transplantation criteria, but for patients with MELD scores <10, the 5-year OS was better after LR than after LT [17]. Additionally, the patients who undergo LT in this study have more advanced cirrhosis, including Child B liver function and portal hypertension; the characteristics of which are not always indications for LR, and this might have been another reason for this discrepancy [41]. However, advanced cirrhosis was defined functionally and not histologically in those studies. For patients with histologically severe cirrhosis and Child A liver function, it remains unclear whether LR or LT should be performed. Using the Laennec staging system in which the cirrhosis of Child A patients is divided into mild (F4A), moderate (F4B), and severe (F4C), we retrospectively studied the long-term outcomes of LR and LT in early HCC patients with varying degrees of cirrhosis. Our unpublished data suggest that LR achieved long-term outcomes that were comparable to those of LT in patients with single HCCs of ≤5 cm in non-cirrhotic or mildly cirrhotic backgrounds, but the LR patients achieved worse outcomes when moderate or severe cirrhotic backgrounds were present. Our preliminary data suggest that LR should remain the first choice for early HCC patients with Child A, mild or no cirrhosis, and that LT should be recommended for those patients with moderate or severe cirrhosis.

### LR or RFA?

LR and radiofrequency ablation (RFA) are alternatives to LT for the treatment of early HCC, and whether RFA or LR is the better treatment option has been debated in recent years. Three recent randomized controlled trials (RCTs) compared the long-term outcomes of LR with those of RFA in early HCC patients and arrived at significantly different conclusions. One of these trials reported that LR produced better survival and lower recurrence rates than RFA among patients with HCCs that fulfilled the Milan criteria and that the 5-year OS rates following LR and RFA were 75.7% and 54.8%, respectively (*P* < 0.05) [42]. However, the other two RCTs reported that the long-term outcomes were comparable between the LR and RFA groups for HCCs of 4 to 5 cm and that the 5-year OS rates for both treatments approached 60% to 70% [43,44]. Several retrospective studies have reported that RFA can achieve long-term outcomes that are comparable to those of LR in the treatment of single HCCs of ≤3 cm in patients with cirrhosis [45,46]. Peng and colleagues [47] recently that the efficacy and safety of RFA were better than those of LR among patients with single HCCs ≤2 cm. Particularly in patients with central HCCs (that is, tumors located at least 3 cm away from the liver capsule), the OS and disease-free survival rates were significantly better in the RFA group than in the LR group [48]. Taken together, these results suggest that several factors might account for these discrepancies. First, in the percutaneous RFA procedure, the treatment of tumors that are larger than a single ablative area required repeated ablations. It is difficult to overlay every ablative area precisely in the three-dimensional liver with the guidance of two-dimensional ultrasonography [42]. Furthermore, another factor that affects the survival of HCC patients is related to underlying liver cirrhosis [20,25,26,49]. Patients undergoing RFA are often cirrhotic or have Child B cirrhosis and are thus typically not good candidates for LR due to the sacrifice of additional normal liver tissue and blood loss, which can induce a greater number of complications and negatively affect treatment outcome [50].

Tumor size and underlying cirrhosis obviously play important roles in determining the treatment outcomes. Many guidelines for the treatment of HCC recommend LR and RFA as alternatives for patients with good liver function, HCCs ≤3 cm and no more than three nodules [12,51,52]. The severity of cirrhosis, tumor sizes, tumor locations, and technical factors should be considered when selecting surgical modalities. For patients with HCCs ≤3 cm, deep tumor locations, and moderate or severe cirrhosis, RFA is a better choice of treatment [12,52-54] because it preserves more liver parenchyma and has a lower risk of postoperative complications [55]. Due to the adverse effects of cirrhosis on the long-term outcomes of HCC, LT is still considered to be the best treatment strategy for patients with moderate or severe cirrhosis if the transplantation criteria are met. RFA can be used as a temporary treatment during the waiting period for LT [54]. For patients with HCCs ≤3 cm without cirrhosis or with mild cirrhosis, RFA and LR can both be valid treatment choices depending on tumor location and technical feasibility.

### Anatomic resection or non-anatomic resection?

LR includes anatomic resection (AR) and non-anatomic resection (NAR). AR is defined as the systematic removal of a hepatic segment confined by tumor-bearing portal tributaries in accordance with Couinaud’s system, whereas NAR is defined as the removal of the tumor with an adequate margin irrespective of the segments.

Debate remains regarding whether AR or NAR should be performed for HCC. Most recurrences that occur in the remnant liver originate from the primary tumor via microscopic vascular invasion and peripheral spread along the portal venous tributaries, which are the most commonly reported risk factors that are associated with poor prognoses [56-58]. Therefore, AR is advocated for HCC treatment to eradicate both the tumors and potential microscopic metastases. Some studies have indicated that the long-term outcomes of AR groups are significantly better than those of NAR groups [59-63]. In contrast, other studies have failed to demonstrate the survival benefits of AR [64-69]. These investigators preferred to perform NAR to preserve larger volumes of functional liver parenchyma in patients with cirrhosis and limited hepatic functional reserves. This preservation decreases the incidence of postoperative liver failure and thus improves long-term outcomes suggesting that the removal of the tumor and the preservation of the liver parenchyma are equally important in such patients. Notably, the major causes of death in these studies included not only recurrence but also progressive liver insufficiency due to advanced cirrhosis because regeneration was impaired, and the normalization of liver function was slow or did not occur at all. Two recent meta-analyses of observational studies compared the long-term outcomes of AR and NAR and reported conflicting results [70,71]. Notably, the assessed studies were not all RCTs, and underlying cirrhosis was more common in the NAR patients who exhibited more advanced liver dysfunction than did the AR patients [54]. A meta-regression approach was utilized to explain this inconsistency [72]. The clearest difference between the patients who underwent AR and NAR was the differential prevalence of cirrhosis as shown in Table 2. This difference indicated a potential selection bias that would have resulted in the long-term outcomes of the HCC patients who underwent AR to appear superior to those of the patients who underwent NAR because the poorer liver function reserve of the latter group significantly affected the prognoses [72]. Theoretically, AR can remove the entire portal venous drainage of the involved segment and provide optimal operative clearance; however, the severity of cirrhosis might play a more important role than the type of resection in long-term survival of HCC, and preservation of the liver parenchyma in patients with cirrhosis should be prioritized over major resection [73]. Some other researchers have recommended that AR should be performed for HCC in patients without cirrhosis and that NAR has proven its long-term efficacy for the treatment of HCC patients with cirrhosis [64,65]. The optimal resection for each patient should be chosen based on liver function, liver functional reserve, and the severity of cirrhosis. For HCC patients with moderate or severe cirrhosis, LT should be the first choice if the tumors fulfill LT criteria. For those tumors that do not meet the LT criteria but are technically resectable, NAR might be an alternative treatment modality. AR should be recommended for HCC patients without cirrhosis or with mild cirrhosis.

**Table 2:** **Comparative studies of survival following AR versus NAR in HCC patients with cirrhosis**

**Authors**	**Type of resection**	** Patients (** ***n*** **)**	**Cirrhosis (%)**	**5-year survival (%)**	*** P*** **value**
Hasegawa *et al*. [59]	AR	156	32.1	66.0	0.01
	NAR	54	57.4	35.0	
Kaibori *et al*. [65]	AR	34	29.4	53.7	0.717
	NAR	213	54.0	52.5	
Wakai *et al*. [61]	AR	95	46.3	67.0	0.036
	NAR	63	66.7	59.0	
Yamashita *et al*. [64]	AR	201	48.8	76.0	NS
	NAR	120	68.3	74.0	
Ueno *et al*. [67]	AR	52	51.9	63.0	0.19
	NAR	64	67.2	58.0	
Eguchi *et al*. [60]	AR	2267	NA	65.5	0.053
	NAR	3514	NA	62.4	
Tanaka *et al*. [66]	AR	83	38.6	54.0	0.34
	NAR	42	52.4	61.0	
Nanashima *et al*. [68]	AR	49	38.8	55.0	NS
	NAR	64	42.2	66.0	
Kamiyama *et al*. [63]	AR	152	23.7	83.0	<0.001
	NAR	133	47.4	65.3	
Kang *et al*. [69]	AR	146	54.8	48.0	0.762
	NAR	21	76.2	40.0	

AR, anatomic resection; NAR, non-anatomic resection; NA, not available; NS, not significant.

## Conclusions

Most cases of HCC are associated with cirrhosis, and the histological severity of cirrhosis varies widely among patients with Child A liver function. The histological severity of cirrhosis is an important adverse factor that affects the long-term outcomes of LR. It is important to recognize that the presence of underlying cirrhosis is critically important for the determination of treatment options. Therefore, further studies of the effects of the histological severity of cirrhosis on the long-term outcomes of surgical modalities are needed to achieve the best surgical outcomes for HCC patients.

Several issues remain to be resolved. First, although the histological severity of cirrhosis can be accurately evaluated by liver biopsy, this invasive procedure might cause new morbidities in patients with liver-diseased patients. A non-invasive method based on imaging technology needs to be developed for the staging of the severity of cirrhosis. Second, a greater number of well-designed RCTs are needed to confirm the value of incorporating the histological severity of cirrhosis into the selection of the appropriate surgical modality for the treatment of HCC.
